# Biomimetic Carbon Sequestration and Cyanate Detoxification Using Heat-Purified Carbonic Anhydrase from *Sulfurihydrogenibium yellowstonense*

**DOI:** 10.3390/biomimetics8040365

**Published:** 2023-08-14

**Authors:** Chia-Jung Hsieh, Chia-Jung Hu, Chi-Yang Yu

**Affiliations:** 1 Department of Chemical Engineering and Biotechnology, Tatung University, Taipei 104327, Taiwan; cc1235896te@gmail.com; 2 Department of Mechanical and Materials Engineering, Tatung University, Taipei 104327, Taiwan; cjhu@ttu.edu.tw

**Keywords:** carbonic anhydrase, carbon capture and sequestration, cyanase, cyanate, *Sulfurihydrogenibium yellowstonense*

## Abstract

The reaction condition for purifying carbonic anhydrase from *Sulfurihydrogenibium yellowstonense* (SspCA) by direct heating without prior cell lysis was optimized; heating at 70 °C for 5 min resulted in the highest total activity of 23,460 WAU (Wilbur–Anderson unit) from a 50 mL culture. Heat-purified SspCA was examined for its capability to increase the rate of the mineralization of CO_2_; compared with an uncatalyzed control, the onset time of CaCO_3_ formation was shortened by up to 71%. Cyanase can be used to degrade toxic cyanate; however, one of the limitations of this biomimetic process is that the reaction needs HCO_3_^−^ as a substrate. Heat-purified SspCA was combined with heat-purified cyanase from *Thermomyces lanuginosus* to alleviate the HCO_3_^−^ dependence; in industrial wastewater, the HCO_3_^−^ required was reduced by 50% when 0.75 WAU of SspCA was added. Heat-purified SspCA is stable at 4 °C; 88% of the initial activity was retained for up to five weeks. Partially purified SspCA can be obtained with ease and applied to a variety of applications.

## 1. Introduction

Carbonic anhydrase (CA, EC 4.2.1.1) catalyzes the hydration of CO_2_ (CO_2_ + H_2_O ↔ HCO_3_^−^ + H^+^), in which aqueous CO_2_ is converted to bicarbonate with the concomitant release of a proton [1]. CA is vital to organisms because it maintains the pH balance by bicarbonate buffering and speeds up CO2 transport to meet the demand of CO2-related metabolic pathways [2].

CA is crucial for carbon capture and sequestration (CCS). CO_2_ adsorption is an important method in CCS. CA has been used to improve the slow CO_2_ adsorption kinetics of the capture solvents [3,4], and thus the costs of equipment and operation are decreased. CA has also been applied to accelerate the slow reaction kinetics of another important CCS method, the mineralization of atmospheric carbon, in which the bicarbonate resulting from the CO_2_ hydration is further converted to mineral carbonates by reacting with metal ions such as Ca^2+^, Mg^2+^, and Fe^2+^ under an alkaline pH [5].

In addition to its applications in CCS, CA can facilitate biomimetic cyanate degradation by coupling with cyanase (Cyn, EC 4.2.1.104). Cyanides are widely used in the agrochemical industry and precious metal extraction industry [6]; these compounds are extremely toxic and recalcitrant to biodegradation. Chemical treatments of cyanide-containing wastes often result in a large amount of less toxic cyanate, a derivative of cyanide after oxidation. Because cyanate can be used as pesticide and fungicide [7], it is sometimes found in the environment. Using bicarbonate as a substrate, cyanase can detoxify cyanate to harmless CO_2_ and ammonia (OCN^−^ + HCO_3_^−^ + 2H^+^ ↔ NH_3_ + 2CO_2_). The dependence on bicarbonate for cyanate degradation can be decreased by coupling Cyn with CA, which provides the necessary bicarbonate via CO_2_ hydration. In turn, the consumed CO_2_ is replenished by Cyn. An enzymatic system composed of Cyn and CA (both from *Thermomyces lanuginosus*) reduces the bicarbonate dependence for cyanate degradation by 80% when compared with only Cyn [8].

In this work, we examined the feasibility of purifying CA from thermophilic *Sulfurihydrogenibium yellowstonense* (SspCA) using a one-step heating process, in which cell lysis and precipitation of constituent proteins are combined. SspCA is known for its exceptional thermal stability; more than half of its original activity was retained even after incubation at 100 °C for 3 h [9]. For carbon sequestration, the enzymes often need to be thermally stable; for instance, a CA modified by directed evolution was used for the carbon capture from flue gas emitted by a coal-fired power plant at 87–100 °C [3]. Besides its exceptional thermal stability, SspCA also possesses high activity, with a k_cat_ of 9.35 × 10^5^ s^−1^ [10]. SspCA is also alkali-stable (active at pH 9.6), which is important for carbon mineralization because the reaction is favored at an alkaline pH [5]. The reaction condition of one-step heating for purifying SspCA was optimized and the results were compared with conventional methods. The feasibility of biomimetic CCS using heat-purified SspCA was examined. In addition, we performed cyanate degradation by coupling SspCA with cyanase from the thermophilic fungus *T. lanuginosus* (TlCyn), which was also purified via simple one-step heating.

## 2. Materials and Methods

### 2.1. Bacterial Strains and Vector Construction

*E. coli* DH5α was used for DNA manipulation and *E. coli* BL21(DE3) was used for expressing the recombinant enzyme. The SspCA gene (NCBI Reference Sequence WP_012459296.1) without the signal peptide was synthesized by Trade Wind Biotech (Taipei, Taiwan) and inserted into a pET-28a(+) vector between the *Nde*I and *Xho*I restriction sites (pET-28a(+)-SspCA). The recombinant SspCA carries an N-terminal (His)_6_-tag as a result of an additional stop codon before the *Xho*I site. For TlCyn cloning, the gene was degenerated from the amino acid sequence of 6XGT_A from the Protein Data Bank; the commercially synthesized gene was also inserted into a pET-28a(+) vector using the *Nde*I and *Xho*I restriction sites. The recombinant TlCyn has an N-terminal (His)_6_-tag because a stop codon was placed before the *Xho*I site.

### 2.2. Expression and Purification of SspCA

The transformants harboring the pET-28a(+)-SspCA were cultured at 37 °C in TB medium with 50 μg/mL of kanamycin. When OD_600_ reached 0.6–0.8, the expression of SspCA was induced by adding 0.1 mM IPTG; 0.5 mM ZnSO_4_ was also added as a source of Zn^2+^, which is required for the enzyme activity. After the culture was further incubated for 8 h at 28 °C, the cells were collected after centrifugation at 8000× *g* for 30 min at 4 °C. To purify SspCA by heating, for every gram of pellet, 5 mL of the storage buffer (20 mM Tris-HCl, pH 8.3) was used to resuspend the cells. The suspension was heated at 70, 80, and 90 °C for 5, 10, 15, and 20 min, followed by centrifugation at 13,000× *g* for 5 min, and finally the supernatant was collected as heat-purified SspCA using a one-step process.

For comparison with the conventional two-step heat purification, the cells were lysed by sonication on ice before heating using a Q125 dismembrator (from QSONICA, Newtown, CT, USA); the setting was 20 min at a 20% amplitude (3 s pulse on and 12 s pulse off). The lysate was heated at 70 °C for 5 min, followed by centrifugation at 13,000× *g* for 5 min, and then the supernatant was collected as heat-purified SspCA using the two-step process.

Also, for the purpose of comparison, the cell pellet was lysed first with the BugBuster protein extraction reagent (from Merck, Darmstadt, Germany), and then purified with a Ni-NTA column. For every gram of cell pellet, 5 mL of the BugBuster protein extraction reagent supplemented with Benzonase Nuclease and rLysozyme (both were purchased from SIGMA, St. Louis, MO, USA) was added; the suspension was then incubated at room temperature for 20 min under gentle mixing. The cell debris was removed by centrifugation at 8000× *g* for 20 min under refrigeration, and then the lysate was subjected to purification using a Ni-NTA His-Bind Resin Chromatography kit (also from Merck) according to the standard protocol. Eluted SspCA was desalted against the storage buffer using a PD-10 column (from SIGMA). The protein concentration was measured using the Bradford assay with BSA standards.

### 2.3. Expression and Purification of TlCyn

The transformants carrying the pET-28a(+)-TlCyn were cultivated at 37 °C in LB medium containing 50 μg/mL of kanamycin; protein expression was induced with 0.8 mM IPTG when OD_600_ reached 0.5–0.7. The cell pellet was collected after further incubation for 8 h at 25 °C. To purify TlCyn by heating, for every gram of pellet, 5 mL of the storage buffer (20 mM Tris-HCl, pH 8.3) was used to resuspend the cells. The suspension was heated at 60 °C for 5 min, followed by centrifugation at 13,000× *g* for 5 min, and finally the supernatant was collected as heat-purified TlCyn.

### 2.4. Activity Assays

The hydratase activity of SspCA was assayed according to the method reported by Capasso et al. [9]. Bromothymol blue, an indicator, was used to track the pH change during the conversion of CO_2_ to bicarbonate. The CO_2_-saturated water was prepared by bubbling CO_2_ into 100 mL of deionized water chilled in an ice-water bath for 3 h. Ten to fifty microliters of the enzyme solution were added to a test tube containing 1 mL of 25 mM Tris-HCl, pH = 8.3, with bromothymol blue already chilled in an ice-water bath. The reaction was initiated by adding 1 mL of the CO_2_-saturated water to the test tube and, immediately, a stopwatch was started. Using 25 mM Tris-HCl, pH = 6.3, with bromothymol blue as a color reference, the time required for the mixture to change from blue (pH = 8.3) to yellow (pH = 6.3) was recorded. The time required for the color change in an uncatalyzed reaction was also recorded by replacing the enzyme solution with an equivalent volume of storage buffer. The activity was reported in Wilbur–Anderson units (WAU), which is defined as (*T*_0_ − *T*)/*T*; *T*_0_ and *T* are the time for the pH to decrease from 8.3 to 6.3 in uncatalyzed and catalyzed reactions, respectively. SspCA can also be assayed for its esterase activity, which was determined at room temperature using *p*-nitrophenylacetate (*p*-NpA) as the substrate. Ten microliters of enzyme solution were added to a mixture containing 0.3 mL of freshly prepared 3 mM *p*-NpA and 0.7 mL of 15 mM Tris sulfate, pH 7.6, and then A_348_ was monitored for 5 min with a Jasco V-550 spectrophotometer. One unit is defined as an increase of 0.03 at A_348_ in 5 min; the increase in A_348_ was corrected against an uncatalyzed reaction.

The cyanase activity assay was modified from the method reported by Ranjan et al. [11]. In a microcentrifuge tube, 0.5 mL of 50 mM Tris-HCl, pH 8; 0.2 mL of 10 mM KOCN; and 0.2 mL of 15 mM NaHCO_3_ were added and vortexed. The reaction was initiated by adding 0.1 mL of 3 µg/mL of cyanase and the mixture was incubated at 60 °C for 10 min. One milliliter of Nessler’s reagent was added to the reaction mixture to stop the reaction, and then A_420_ was measured using a JASCO V-550 spectrophotometer at an ambient temperature. The amount of ammonium produced by the cyanase reaction was determined from a calibration curve constructed with NH_4_Cl standards. One unit (U) of activity is defined as the production of 1 μmol of ammonium per minute.

### 2.5. CO_2_ Sequestration

The CO_2_ sequestration was performed according to the method reported by Jo et al. [12]. In a disposable cuvette, 50 µL of SspCA solution was mixed with 450 µL of 1 M Tris containing 20 mM CaCl_2_, pH = 11. Five hundred microliters of CO_2_-saturated water prepared at 30 °C was added to initiate the sequestration reaction, which was monitored by measuring A_600_ using a thermostated spectrophotometer (JASCO V-550) at 30 °C. Instead of the 0 °C used for the hydratase activity assay (Section 2.4), CO_2_-saturated water was prepared at 30 °C because such a temperature is closer to the operation temperature of most CO_2_-capturing facilities [13,14]. For characterizing the precipitated CaCO_3_, the precipitate was collected by filtering it through a 0.45 μm membrane filter, and then dried at 70 °C overnight in an oven before SEM and XRD analysis. The SEM image was obtained with a Hitachi SU8000 scanning electron microscope. The XRD patterns were determined with a Bruker D2 PHASER diffractometer.

### 2.6. Cyanate Degradation by TlCyn and SspCA

In a microcentrifuge tube, 0.5 mL of 50 mM Tris-HCl, pH 8.0; 0.2 mL of 20 mM KOCN; and 0.2 mL of NaHCO_3_ (2.5, 5, 7.5, 10, 12.5, and 15 mM) were added and vortexed. In a water bath set at 60 °C, 50 µL of TlCyn and 50 µL of SspCA were added to the mixture, followed by incubation for 10 min with shaking at 100 rpm. The ammonium released from cyanate degradation was determined with the procedure described in Section 2.3. Industrial wastewater was collected from the electric motor factory of the Tatung Company (New Taipei City, Taiwan) and the metal ions contained within were determined with an ICP optical emission spectrometer. In order to study the possible effects from highly polluted wastewater, artificial wastewater was prepared by spiking deionized water with 13.2 µM Cd^2+^, 0.24 mM Pb^2+^, 0.48 mM Cr^6+^, 0.86 mM Ni^2+^, and 1.2 µM Hg^2+^; these concentrations are five-fold the effluent standards of electroplating wastewater specified by the Environmental Protection Administration of Taiwan [15]. To study the effect of the matrix on degradation, 50 mM Tris-HCl was replaced with either industrial or artificial wastewater.

### 2.7. Statistical Analysis

All tests were performed in triplicate. The data were reported as mean ± SD. Analysis of variance (ANOVA) was carried out using SPSS software (SPSS Inc., Chicago, IL, USA) to analyze differences between results.

## 3. Results and Discussion

### 3.1. Purification of SspCA by Heating

The recombinant SspCA is composed of 245 amino acids; as calculated using the Compute pI/Mw tool from the Expasy website [16], the enzyme has a molecular weight of 28.3 kDa and a theoretical pI of 9.23. The molecular weight was confirmed by SDS-PAGE using Ni-NTA column-purified enzyme.

The results from the heat-purification are listed in Table S1. The purity was not affected by the heating time significantly, suggesting that the precipitation of the constituent proteins was completed within the first 10 min. The purities obtained from 70 and 90 °C were lower than those from 80 °C; the highest purity of 64% was observed after 10 min heating at 80 °C. A longer heating time resulted in a lower total activity at 80 and 90 °C; however, a similar effect was not observed at 70 °C. Moreover, the total activities obtained from 90 °C were far lower than those from 70 and 80 °C. These observations prompted us to examine the thermal stability of column-purified SspCA by assaying its hydratase activity every 30 min; after 150 min, the enzyme retained 68, 60, and 49% of the original activity at 70, 80, and 90 °C, respectively. The results from the thermal stability study indicated that the thermal deactivation of SspCA may partly explain the low total activity obtained from 90 °C. The total protein was not affected significantly by heating time; however, the total protein obtained from 80 and 90 °C was much less than that obtained from 70 °C, suggesting that protein precipitation was more effective at 80 and 90 °C. Higher specific activities were observed at 80 °C due to the relative high activity and low total protein at this temperature. The optimal condition was heating at 70 °C for 5 min because of its highest total activity; this condition was used for later purification. Recombinant SspCA purified by a two-step heating process has been reported [17], in which the host cells were lysed first by sonication, followed by heating of the lysate at 70 °C for 30 min; the total protein was 100 mg with a purity of 60% from a 2-L culture. Compared with the optimal condition of our one-step process (heating at 70 °C for 5 min), after simple calculation (total protein × purity), the recombinant SspCA obtained from the one-step process was 2.7-fold higher than that from the two-step process.

The effect of a prior cell lysis on the purification performance was studied (Table 1); SspCA was also purified with the widely used Ni-NTA column for a thorough comparison. As expected, the highest purification and the lowest total protein were obtained with the Ni-NTA column because of its high specificity; the purity of SspCA reached 96%. The yield of the one-step heating was very close to that of the Ni-NTA column but slightly lower than that of the two-step heating, suggesting that the purification performance of the one-step process was not significantly impeded due to the omission of a prior cell lysis step.

### 3.2. CO_2_ Sequestration in CaCO_3_

Column-purified and heat-purified SspCA were used to increase the reaction rate of the conversion of CO_2_ to CaCO_3_ (Figure S1). The onset time of CaCO_3_ precipitation, defined as the time when the highest ΔA_600_ per second is observed, is listed in Table 2. As expected, the addition of a higher concentration of column-purified SspCA resulted in a shorter onset time; the onset time of the control (no enzyme was added) was decreased by 28, 38, and 57% when 50 µL of 250, 300, and 350 µg/mL of heat-purified SspCA was added, respectively. When the same concentration (estimated from the 41% purity of heating at 70 °C for 5 min in Table S1) of heat-purified SspCA was added, to our surprise, the onset time was shorter than that of the column-purified SspCA; the onset time of the control was decreased by 57, 60, and 71% when 50 µL of 250, 300, and 350 µg/mL of heat-purified SspCA was added, respectively. One possible explanation for the faster CaCO_3_ precipitation rate of the heat-purified SspCA is that the protein impurities may contain amino acid sequences that enhance CaCO_3_ precipitation; it has been shown that peptide derived from naturally occurring proteins [18] and aspartate-rich synthetic peptides [19] are capable of increasing the CaCO_3_ formation rate. In Figure S1, except for the negative control, a plateau was reached for all the curves, suggesting the possible inactivation of SspCA under pH 11 or the depletion of dissolved CO_2_. We further examined the pH stability of SspCA by incubating the enzyme in a buffer system composed of 150 mM glycine, 150 mM H_3_PO_4_, and 150 mM Tris-base with the pH adjusted to 9, 10, and 11 for 30 min, and SspCA retained 84%, 83%, and 69% of its hydratase activity, respectively. Based on these results, the plateaus observed within 300 s in Figure S1 are more likely due to the depletion of dissolved CO_2_. The XRD analysis showed that the precipitate formed without the enzyme (black trace, Figure S2) and the column-purified SspCA (blue trace, Figure S2) were quite similar; the precipitates were composed mainly of spherical vaterite and some rhombohedral calcite, which is also evident from the SEM images (Figure S3A,B). From both XRD data (red trace, Figure S2) and SEM images (Figure S3C), the precipitate formed by heat-purified SspCA contained more rhombohedral calcite when compared with those from the control and column-purified SspCA. The different CaCO_3_ morphology resulting from heat-purified SspCA could be related to the protein impurities; the macromolecules in the biomineralization process can affect the growth morphology of crystals [20].

### 3.3. Cyanate Degradation Using TlCyn and SspCA

TlCyn is ideal for detoxifying cyanate under harsh conditions because of its stability and heavy metal tolerance [11]. In order to study the influence of the matrix, degradation was also carried out in industrial wastewater and artificial wastewater in addition to the buffer.

**Figure 1:**
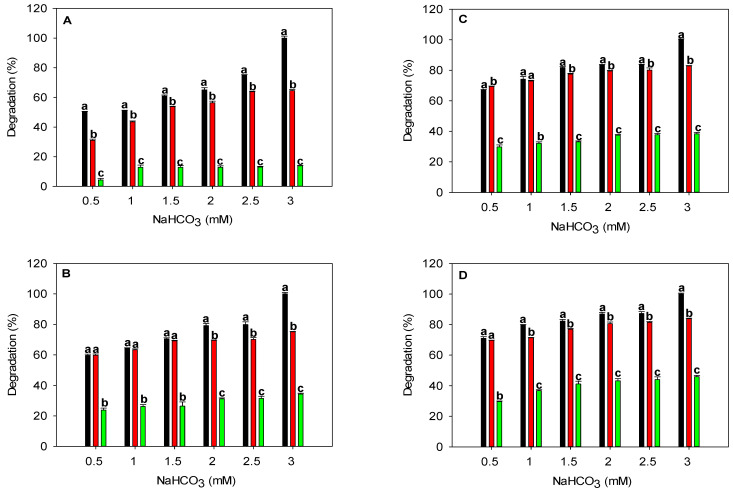
Degradation of cyanate in different matrices using heat-purified enzymes. The activity of the SspCA added was 0 (**A**), 0.5 (**B**), 0.75 (**C**), and 1 (**D**) WAU; the activity of the TlCyn added was 0.03 U. Degradation was performed in 50 mM Tris-HCl, pH 8 (black); industrial wastewater (red); and artificial wastewater (green). The value of 100% was defined as the cyanate degradation observed with 3 mM NaHCO_3_ in the buffer. Different letters at the same NaHCO_3_ concentration indicate a significant difference at *p* < 0.05.

The cyanate degradation in different matrices is shown in Figure 1. For cyanate degradation in the buffer with TlCyn only (Figure 1A, black bars), the bicarbonate dependence is clearly observed because the degradation increases with the increasing NaHCO_3_ concentration. In the buffer (Figure 1, black bars), with 0.5 mM NaHCO_3_, the cyanate degradation increased from 50.2% of the control (TlCyn only, Figure 1A) to 59.7%, 66.9%, and 71% when 0.5, 0.75, and 1 WAU of SspCA was present (Figure 1B,C), respectively. The bicarbonate dependence diminishes as SspCA activity increases. For easier analysis of the bicarbonate dependence, we calculated the difference in cyanate degradation (DCD, listed in Table 3), which is defined as the cyanate degradation in percentage using 0.5 mM NaHCO_3_ subtracted from that using 3 mM NaHCO_3_. Using cyanate degradation in the buffer as an example, a DCD value of 49.8%, 40.3%, 33.1%, and 29% (third row in Table 3) was obtained for the control, 0.5, 0.75, and 1 WAU of SspCA, respectively, by subtracting 50.2%, 59.7%, 66.9%, and 71% from 100% of the 3 mM NaHCO_3_. A lower DCD value indicates that the reaction requires less HCO_3_^−^, presumably because the added SspCA provides bicarbonate by converting dissolved CO_2_ in addition to the initial 0.5 mM NaHCO_3_. In the buffer, the DCD decreased as SspCA activity increased, clearly showing that the addition of SspCA decreased the dependence on HCO_3_^−^ of cyanate degradation. A similar improvement in the HCO_3_^−^ dependence was also observed in the industrial wastewater up to 0.75 WAU; the HCO_3_^−^ required was reduced by 50% because the cyanate degradation using 1.5 mM NaHCO_3_ was very close to that using 3 mM NaHCO_3_ when 0.75 WAU of SspCA was added (Figure 1C). In the artificial wastewater, using TlCyn alone (Figure 1A), the cyanate degradation was significantly lower than that in other matrices; suggesting that the enzyme was inhibited. It has been shown that 89%, 97%, 89%, 97%, and 89% of the initial activity remained after incubation with 5 mM Cr^6+^, Cd^2+^, Hg^2+^, Ni^2+^, and Pb^2+^, respectively [11]. The metal ions present in the artificial wastewater should not cause significant inhibition on TlCyn because the concentrations we used are much lower than the reported 5 mM; other components such as anions may lead to the observed inhibition. In the artificial wastewater, the addition of SspCA did not improve the dependence on HCO_3_^−^, as the DCD value was not decreased (Table 3). We further examined the effects of metal ions present in the artificial wastewater at 5 mM (the same as the concentration used for the reported TlCyn inhibition study) on the activity of SspCA; the residual activity after 30 min of incubation was 33, 19, 34, 42, and 22 for Ni^2+^, Hg^2+^, Cd^2+^, Cr^6+^, and Pb^2+^, respectively. Our results indicate that SspCA is much more susceptible to the inhibition imposed by the tested metal ions than TlCyn, which partly explains why SspCA did not improve the dependence on HCO_3_^−^.

### 3.4. Storage Stability

**Figure 2:**
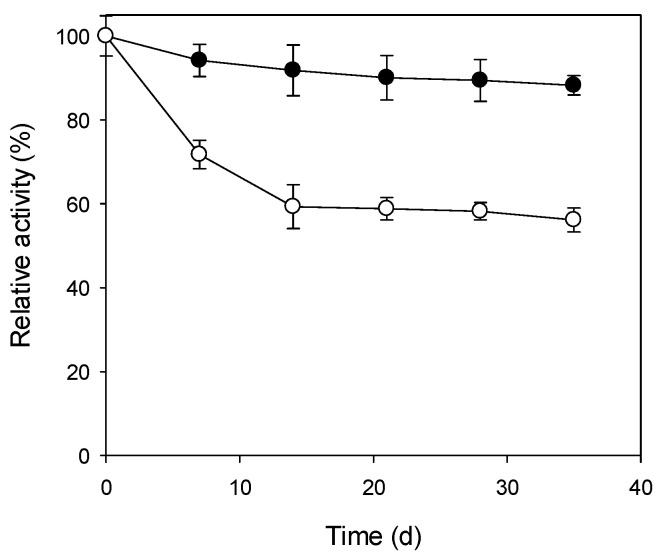
Storage stability of heat-purified SspCA at 4 and 25 °C. Solid circle: 4 °C; open circle: 25 °C. Initial activities were defined as 100%. The residual activity was monitored weekly using the CO_2_ hydratase activity assay.

The activity of heat-purified SspCA showed a slow decrease and retained 88% of the initial value at 4 °C after five weeks (Figure 2), indicating that the enzyme is rather stable at this temperature. When stored at 25 °C, the heat-purified SspCA demonstrated a significant loss in activity, with 59% of the initial value remaining after the first two weeks; however, the enzyme lost almost no activity within the next three weeks and retained 56% of the initial value. The storage stability of heat-purified SspCA is very similar to that of its column-purified counterpart at both temperatures, suggesting that the impurities resulting from heat purification do not affect the storage stability.

## 4. Conclusions

The feasibility of purifying thermostable SspCA using a simple one-step heating process was demonstrated and the reaction condition was optimized. Under the optimal 5 min heating at 70 °C, the purification performance was not too different from a two-step process with a prior cell lysis step. Heat-purified SspCA successfully enhanced the reaction rate of CO_2_ sequestration in CaCO_3_ and alleviated the HCO_3_^−^ dependence of cyanate detoxification. Heat-purified SspCA also showed reasonable storage stability up to five weeks. The inhibition of SspCA by metal ions can be mitigated by techniques such as immobilization, rational design, and directed evolution, and hence the cyanate degradation efficiency of the enzymatic system could be improved. For an enzymatic process that is normally performed at large scale, like the CO_2_ sequestration and the cyanate detoxification we reported, the cost of the enzyme is always crucial. Compared with a commonly used protein purification process such as cell lysis followed by ammonium sulfate precipitation or chromatography, the cost of the one-step heat purification should be much lower because it does not require specialized reagents nor equipment. The one-step process should be applicable to other thermostable proteins for applications that do not require high purity.

## Figures and Tables

**Table 1:** Comparison of different purification methods for SspCA. The data were obtained from a culture volume of 50 mL.

Purification Method	Total Activity (WAU) ^3^	Total Protein (mg)	Specific Activity (WAU/mg)	Purification (x) ^4^	Yield (%) ^5^
Crude lysate	29,039 ± 4250	85 ± 2.6	342 ± 50	1	100
One-step heating ^1^	23,460 ± 5974	10 ± 0.2	2441 ± 621	7.2	81
Two-step heating ^2^	27,712 ± 3371	13 ± 0.3	2061 ± 251	6.0	95
Ni-NTA column	23,806 ± 4043	6 ± 0.2	3911 ± 664	12	82

^1^ Heating was performed at 70 °C for 5 min. ^2^ Heating was performed at 70 °C for 5 min after sonication. ^3^ Wilbur–Anderson unit. ^4^ Purification = the specific activity of each method/that of the lysate. ^5^ Yield = (the total activity of each method/that of the lysate) × 100.

**Table 2:** The onset time of CaCO_3_ precipitation.

		Purification Method					
		Ni-NTA Column			Heating		
SspCA (µg/mL)	Control	250	300	350	250	300	350
Onset time (s)	93 ± 15	67 ± 0	58 ± 0	40 ± 0	40 ± 0	37 ± 6	27 ± 6

**Table 3:** Difference in cyanate degradation under different reaction conditions.

	DCD (%) ^1^			
SspCA (WAU)	0	0.5	0.75	1
Buffer	49.8 ± 1.5	40.3 ± 2.3	33.1 ± 0.9	29 ± 2.3
Industrial wastewater	33.6 ± 0.7	15.5 ± 0.8	13.4 ± 0.1	14.1 ± 0.4
Artificial wastewater	9.6 ± 1.1	10.5 ± 1.7	8.6 ± 1.7	15.9 ± 0.8

^1^ Difference in cyanate degradation (DCD) = (cyanate degradation in percentage using 3 mM NaHCO_3_) − (cyanate degradation in percentage using 0.5 mM NaHCO_3_).

## Data Availability

No new data were created or analyzed in this study. Data sharing is not applicable to this article.

## Supplementary Materials

The following supporting information can be downloaded at: https://www.mdpi.com/article/10.3390/biomimetics8040365/s1, Figure S1: Rate of CaCO_3_ precipitation; Figure S2: XRD patterns of precipitated CaCO_3_; Figure S3: SEM images of precipitated CaCO_3_; Table S1: Properties of SspCA purified by heating.
