# Supplementary material for: Biomimetic Carbon Sequestration and Cyanate Detoxification Using Heat-Purified Carbonic Anhydrase from Sulfurihydrogenibium yellowstonense
Source: Biomimetics (Basel). 2023 Aug 14;8(4):365. doi: 10.3390/biomimetics8040365 (PMC10452739; doi:10.3390/biomimetics8040365)
Supplement: Supplementary file 1 [file biomimetics-08-00365-s001.zip › biomimetics-2450460-supplementary.pdf]

Supplementary material

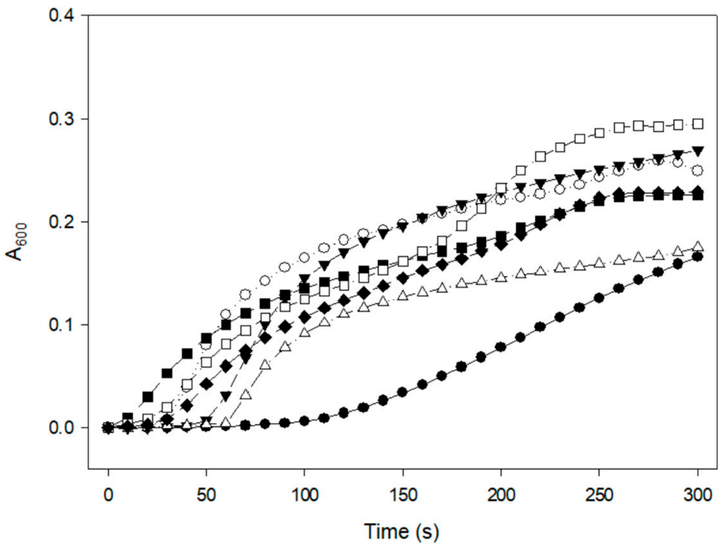

Figure S1. Rate of $CaCO_3$ precipitation. Solid circle: negative control without enzyme; addition of 250 (open triangle), 300 (solid triangle), and 350 (open circle) µg/mL of column-purified SspCA; addition of 250 (solid diamond), 300 (open square), and 350 (solid square) µg/mL of heat-purified SspCA.

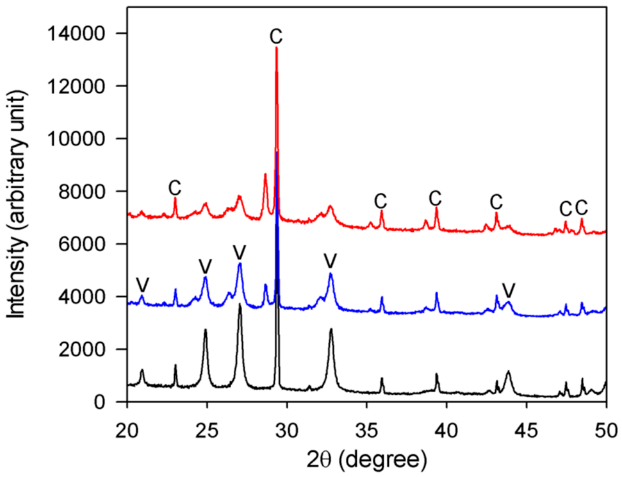

Figure S2. XRD patterns of precipitated $CaCO_3$. C: calcite; V: vaterite. Black: negative control without the addition of enzyme; blue: precipitation catalyzed by Ni-NTA column-purified SspCA; red: precipitation catalyzed by heat-purified SspCA.

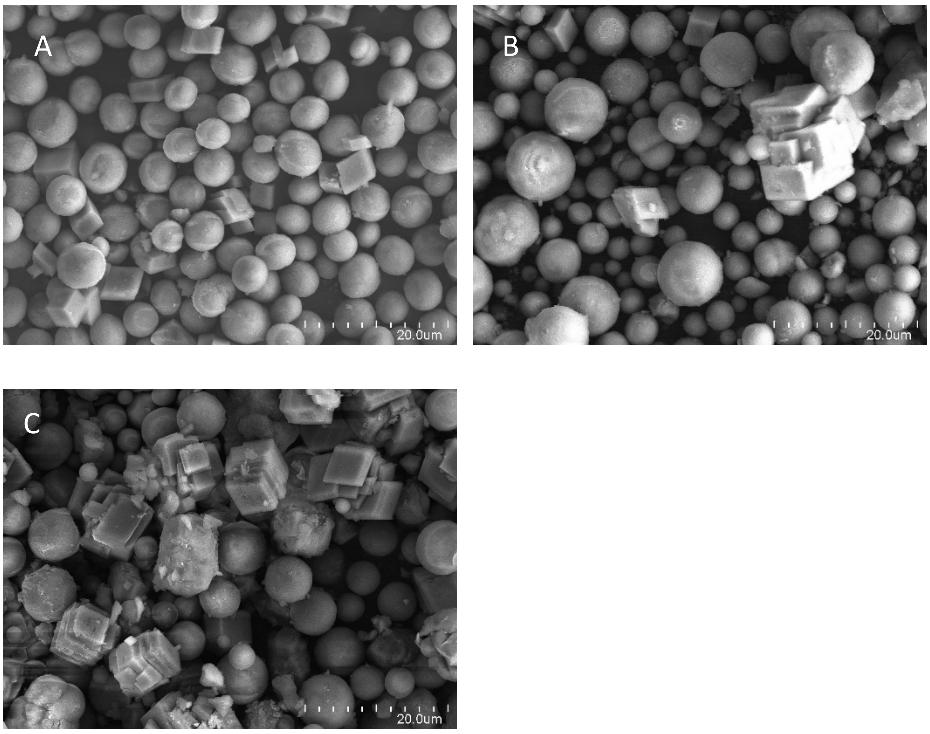

Figure S3. SEM images of precipitated $CaCO_3$. (**A**) Negative control without the addition of enzyme; (**B**) precipitation catalyzed by Ni-NTA column-purified SspCA; (**C**) precipitation catalyzed by heat-purified SspCA.

Table S1. Properties of SspCA purified by heating. The data were obtained from a culture volume of 50 mL.

Temperature (°C)	70				80				90			
Time (min)	5	10	15	20	5	10	15	20	5	10	15	20
Purity (%) [1]	41	51	50	48	60	64	60	61	40	45	43	39
Total activity (WAU)	23460± 5974	18833 ± 3315	20822 ± 3315	18576 ± 5538	19098 ± 2082	20106 ± 341	14953 ± 3192	11720 ± 1768	6844 ± 1420	6429 ± 417	3718 ± 725	2028 ± 330
Total protein (mg)	10 ± 0.2	7 ± 0.1	8 ± 0.04	9 ± 0.7	3 ± 0.3	3 ± 0.4	3 ± 0.2	3 ± 0.4	2 ± 0.3	2 ± 0.4	2 ± 0.3	1 ± 0.1
Specific activity (WAU/mg prot.)	2441 ± 621	2758 ± 485	2470 ± 393	2145 ± 639	5870 ± 640	5949 ± 101	4495 ± 959	3920 ± 591	3334 ± 692	3878 ± 246	2419 ± 471	1435 ± 220

[1] The purity was determined from SDS-PAGE using densitometry.
